# Glial interactions in the formation and plasticity of the corpus callosum

**DOI:** 10.3389/fncel.2025.1690400

**Published:** 2025-11-27

**Authors:** Joanna Czyrska, Marta Marlena Ziętek, Agnieszka Bernat, Silvestre Sampino

**Affiliations:** 1 Department of Experimental Embryology, Institute of Genetics and Animal Biotechnology of the Polish Academy of Sciences, Jastrzębiec, Poland; 2 Laboratory of Photobiology and Molecular Diagnostics, Intercollegiate Faculty of Biotechnology, University of Gdańsk and Medical University of Gdańsk, Gdańsk, Poland

**Keywords:** corpus callosum, neurons, astrocytes, oligodendrocytes, microglia, animal models

## Abstract

The corpus callosum (CC) is the largest interhemispheric commissure in the eutherian brain, enabling inter-hemispheric sensory integration and higher-order cognitive functions. Historically viewed through a neuron- and axon-centric lens, extensive research has established that glial cells (astrocytes, oligodendrocytes, and microglia) are essential regulators of CC ontogenesis. Astrocytic guidepost cells sculpt midline architecture and secrete axonal guidance cues; oligodendrocytes drive callosal axonal maturation and myelination; and microglia regulate their fasciculation and pruning, myelination patterns, and synaptic refinement. In addition to these cell-specific roles, coordinated bidirectional signaling between neurons and glia ensures that axon targeting, maturation, and interhemispheric integration proceed in a precisely orchestrated manner. Disruptions to these glial functions are implicated in congenital and developmental brain pathologies, including malformations and CC agenesis. This review integrates molecular, developmental, and translational insights to provide a comprehensive, mechanistic understanding of glial contributions to CC development and how their dysfunction shapes pathology.

## Introduction

The corpus callosum (CC) constitutes the principal interhemispheric commissure in the brain of placental mammals and is fundamental for coordinating bilateral sensory, motor, and cognitive functions. Although the ontogeny of the CC has been historically attributed to the pathfinding behaviors of cortical projection neurons, early studies have identified non-neuronal glial structures as major players in the formation of the CC (Silver et al., 1982; Wahlsten, 1987). While intrinsic neuronal programs remain critical to this process, growing evidence from the last decades has revealed a more complex and multifactorial mechanism that highlights the vital roles of non-neuronal cell types, particularly glial cells, in orchestrating the sequence of developmental events that establish the correct formation of the CC (Katz et al., 1983; Shu and Richards, 2001; Liu et al., 2023; Gavrish et al., 2024; Rodríguez-Pérez et al., 2024).

Current evidence indicates that astrocytes, oligodendrocytes, and microglia participate actively in callosal formation and remodeling. Astrocytes located at the midline generate structural substrates such as the glial wedge and secrete guidance cues, including Slits and Netrins, which regulate axonal attraction and repulsion during midline crossing (Shu et al., 2003; Gobius et al., 2016). Glial cells also contribute to later stages of callosal development through synaptogenic functions and metabolic support. Oligodendrocyte precursor cells (OPCs) populate the callosal region during embryogenesis and initiate myelination in a tightly regulated, regionally specific, and activity-dependent manner (Simons et al., 2024). This process is governed by paracrine signaling pathways, including Sonic Hedgehog (Shh), PDGF, Wnt/β-catenin, Notch, and thyroid hormone signaling, as well as by neuron-derived ligands such as Neuregulin-1 (Calver et al., 1998; Choudhry et al., 2014; Meyer et al., 2018). Microglia, the resident immune cells of the central nervous system, contribute to axon fasciculation, the elimination of redundant or mistargeted axons, the refinement of synaptic circuits, and the regulation of oligodendrocyte dynamics (Cunningham et al., 2013; Filipello et al., 2018; Irfan et al., 2022; Lawrence et al., 2024).

By comparing the ontogenesis of the CC in humans and rodents, this review highlights both conserved and species-specific mechanisms, contextualizes developmental timelines, anatomical organization, and the spatial dynamics of axonal crossing, with a focus on the emergence of key glial structures such as the glial wedge, midline zipper glia (MZG), indusium griseum (IGG), and subcallosal sling (SCS), and their molecular signaling. Next, we elaborate on the roles of astrocytes, with emphasis on their morphogenetic contributions to neuroanatomic remodeling of the midline during fetal stages, as well as axon-guidance cues molecules (e.g., Slit/Robo, Netrin, Draxin pathways), and regulatory functions in synaptic refinement and metabolic homeostasis. Then, we focus on oligodendrocytes, detailing the sequential phases of OPCs proliferation, migration, and differentiation, and highlighting the influence of signaling networks in regulating callosal myelination in the late phase of callosal maturation. Next, we examine the multifaceted functions of microglia in callosal development, ranging from the regulation of axonal fasciculation and neuronal pruning to the phagocytosis of excess OPCs and modulation of local inflammatory environments. Thereafter, we explore how cellular actions integrate into coherent developmental signaling, highlighting neuron-glia communication pathways as the main actors regulating CC prenatal ontogenesis and postnatal function. Finally, we discuss genetic knockout models and human case studies, which connect disrupted glial signaling to specific callosal pathologies, and outline translational tools and emerging methodologies, such as single-cell transcriptomics, iPSC-derived organoids, and enhancer activity-dependent genetic tools, that enable mechanistic investigations of specific glial functions. By assessing the most recent evidence on the cellular and molecular architecture of glial contributions to CC development, this review serves as a resource for understanding the role of glial cells in the ontogenesis of interhemispheric networks in mammals, as well as the formation and integration of the signaling between glial and neuronal cells in the development and agenesis of the corpus callosum.

## Corpus callosum ontogenesis in human and rodents

The development of the CC follows a conserved sequence of events across mammalian species, although the timing and spatial organization differ between rodents and humans. In humans, the corpus callosum starts forming around gestational week 8 (GW8), and the first axons cross the midline between GW12 and GW20. After birth, the CC continues to mature, primarily through the gradual accumulation of myelin around axons, a process that lasts into adolescence (Paul, 2011; Birnbaum et al., 2020). In mice, callosal axons start crossing the midline during late pregnancy, around embryonic days 17–18 (E17–E18), and this process continues actively during the first 2–3 weeks after birth (Table 1). Despite these temporal differences, the main stages of callosal formation, axon emergence, guidance, and crossing through the midline, and then targeting the opposite hemisphere, remain conserved among placental (e.g., eutherian) mammals (De León Reyes et al., 2020).

**TABLE 1:** Timing of callosal development in humans and mice.

Feature	Human	Mouse
Prenatal timing	GW 8–20 (∼2 months duration)	E15–E18 (∼4 days duration)
Postnatal timing	Extended (∼20–25 years)	Rapid (∼first month postnatal)
Myelination pattern	Gradual (infancy to adulthood)	Rapid (within first month)
Plasticity period	Prolonged, experience-dependent	Short, limited window (∼P30)
Axonal refinement	Extended (childhood/adolescence)	Rapid (within early postnatal weeks)
Neurodevelopmental implications	Sensitive to extended prenatal/postnatal disruptions	Sensitive primarily to brief prenatal disruptions

Glial structures at the midline are critical in guiding axons by secreting signaling molecules and drive neuronal axons through their pathfinding. Astrocytes located in the glial wedge and indusium griseum glia, along with the transient subcallosal sling, create a signaling environment that shapes axon paths. The glial wedge, in particular, secretes Slit proteins that direct axons toward the appropriate crossing zone (Shu et al., 2003; Gobius et al., 2016). At the same time, glial cells from ventral midline regions release Netrin-1, which attracts axons that express the DCC receptor (Kang et al., 2018; Sagi-Dain et al., 2020; Morcom et al., 2021b). The balance between repulsion and attraction guides callosal axons across the midline, while molecules like L1CAM and extracellular matrix proteins promote axon bundling and growth (Gómez et al., 2008; Peotter et al., 2022). Other signaling molecules further fine-tune this process. Ephrin-A and EphA receptors, Semaphorin3C and its receptor Nrp1, and Reelin signaling help organize cortical structure and axon sorting (Hu et al., 2003; Cargnin et al., 2018; Mire et al., 2018; Zhi et al., 2025). Genetic disruption of these systems in animal models, such as knockouts of Netrin-1, DCC, or Slit/Robo, leads to misrouted axons, absent callosal connections, or the formation of Probst bundles, where axons fail to cross the midline and instead run along the ipsilateral hemisphere (Unni et al., 2012; Fothergill et al., 2014; Galichet et al., 2021). During this period, both neurons and glial cells migrate to reshape the midline tissue architecture. Astrocyte-mediated remodeling of the interhemispheric fissure (IHF) is essential to allow axons to bridge the hemispheres (Gobius et al., 2016; Liu et al., 2023). This coordinated sequence of growth, signaling, and structural change appears conserved across placental mammals, reflecting a shared molecular foundation for interhemispheric connectivity mediated by the fetal glia.

## Astrocytes in CC development

**FIGURE 1:**
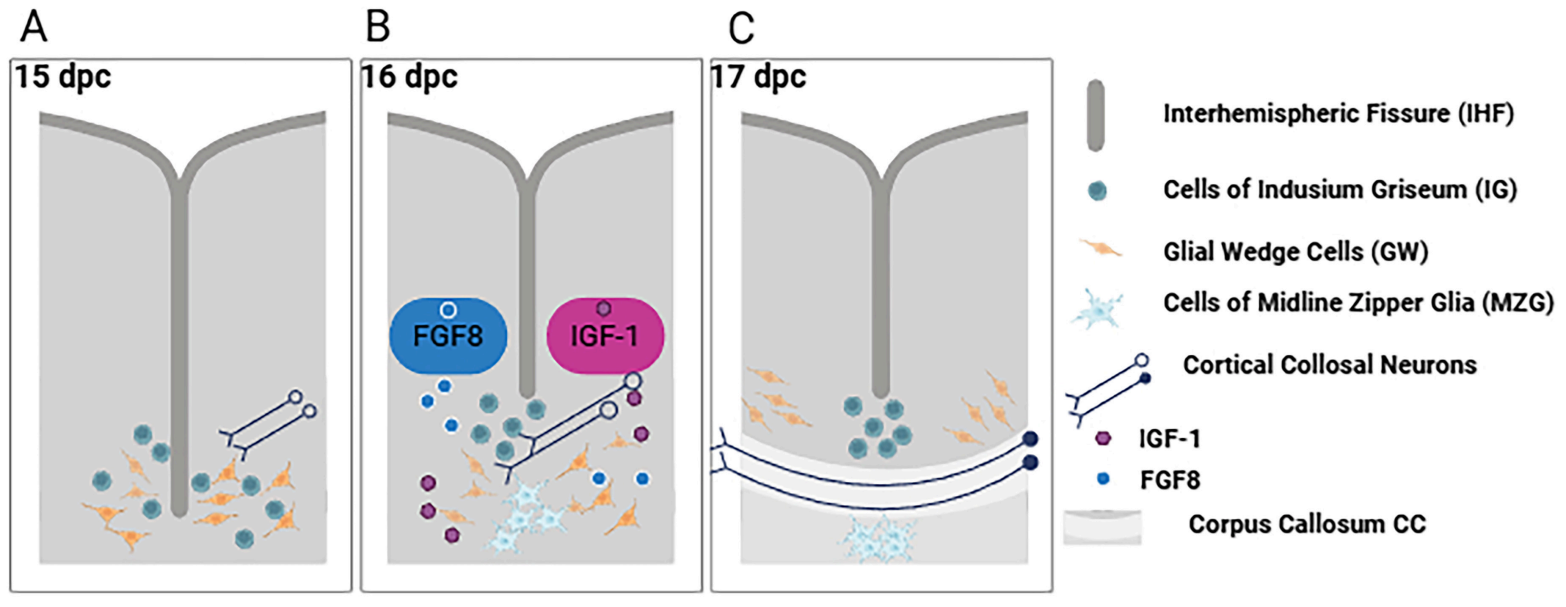
Multi-stage inter-hemispheric fissure remodeling mediated by the glial structures localized at the midline (indusium griseum, midline zipper glia, and glial wedge). **(A)** Midline glial structures change conformation throughout multiple developmental stages and **(B)** secrete growth factors, such as FGF8 and IGF1, which in turn stimulate axon extension toward the midline, **(C)** leading to normal development of the CC. Created in BioRender (https://app.biorender.com/citation/6904bbd3cf3d6e0fe32c2298).

Astrocytes are star-shaped glial cells traditionally known for maintaining homeostasis and supporting neurons. Far from passive support cells, astrocytes play active roles in CC development (Verkhratsky and Nedergaard, 2018; Meyer et al., 2018; Stogsdill et al., 2023). During embryogenesis, specialized astroglial cells at the midline act as navigational guideposts for callosal axons. They form key structures such as the glial wedge and midline “zipper” glia, which secrete guidance factors that drive axon crossing the midline (Shu et al., 2003; Gobius et al., 2016; Verkhratsky and Nedergaard, 2018; Liu et al., 2023). For example, midline astrocytes release Slit proteins that bind Robo receptors on cortical callosal axons, driving them to appropriate trajectories and conveying axonal bundles toward crossing the interhemispheric fissure (Gobius et al., 2016; Liu et al., 2023). This ensures that pioneer axons approach the midline correctly and do not erroneously enter other brain regions. Concurrently, astrocytes produce several trophic and attractants like growth factors (e.g., FGF8, IGF-1) to encourage axon extension toward the midline (Fothergill et al., 2014; Suarez et al., 2014; Figure 1). Disruption of astroglial guideposts has severe consequences: ablating or mispositioning the glial wedge in animal models leads to callosal agenesis, highlighting that the astrocytic guide function is indispensable (Bedeschi et al., 2006; Clegg et al., 2019; Rapti, 2023).

Beyond guidance, astrocytes act as a physical scaffold for migrating neuronal axons. They extend long processes that interdigitate with the axonal growth cones, offering a substrate for inter-neurons connectivity (Meyer-Franke et al., 1999). Astrocytes can also modulate the extracellular environment by secreting extracellular matrix molecules that promote axon adhesion and bundling (Song and Dityatev, 2018; Lagos-Cabré et al., 2020). In these ways, astrocytes ensure that the nascent callosal axons maintain the correct trajectory as the corpus callosum forms. Recent studies have shown that the transformation of radial glia into the so-called midline “zipper” astroglia and their anatomical remodeling in the interhemispheric fissure are crucial steps for callosal axons to cross the midline (Gobius et al., 2016, 2017; Morcom et al., 2021b).

Astrocytes also shuttle nutrients (glucose and lactate, in particular) from blood to neurons and clean up excess neurotransmitters or ions, thereby optimizing the microenvironment for growing axons (Brown et al., 2004; Goursaud et al., 2009; Morrison et al., 2013). Such metabolic support is crucial, as axonal growth and subsequent myelination are energy-demanding processes. This support is mediated by glucose uptake through astrocytic GLUT1 transporters and conversion to lactate, which is then delivered to neurons via monocarboxylate transporters (MCT1 in astrocytes and MCT2 in neurons) to fuel oxidative phosphorylation. Additionally, astrocytes regulate extracellular glutamate and potassium through EAATs and Kir4.1 channels, maintaining ionic balance critical for axonal signaling and growth cone motility. Recent studies suggest that lactate shuttling not only sustains neuronal metabolism but also acts as a signaling molecule promoting axon elongation through NADH-mediated redox signaling and BDNF expression (Mason, 2017; Debernardi et al., 2003; Eid et al., 2019; Yang et al., 2014).

Notably, astrocytes express high levels of transporters for lactate and ions, positioning them as key regulators of energy supply to developing white matter and callosal projections (Fayol et al., 2004; Rinholm et al., 2011; Meyer et al., 2018; Matthiesen et al., 2022).

In later development, astrocytes influence synaptogenesis and circuit refinement in the forming corpus callosum (Levitt, 2003; Clarke and Barres, 2013). They secrete factors such as thrombospondins (TSP-1, -2) and Chordin-like 1 that promote synapse formation among callosal projection neurons (Christopherson et al., 2005; Kucukdereli et al., 2011). Astrocytes also release trophic factors (e.g., brain-derived neurotrophic factor, BDNF) that fortify emerging synaptic connections (Bathina and Das, 2015). Moreover, astrocytes help eliminate weaker synapses by engulfing synaptic debris and releasing signals that activate microglial pruning (*discussed below*). For instance, astrocyte-derived TGF-β induces neurons to express the complement protein C1q, which tags weak synapses for removal by microglia (Stevens et al., 2007). Simultaneously, astrocyte-secreted IL-33 acts on microglia to enhance their engulfment of synapses during developmental remodeling (Vainchtein et al., 2018). This bidirectional role of astrocytes toward neurons and non-neuronal cells ensures that robust interhemispheric synapses persist while redundant ones are pruned, which is a crucial process for efficient inter-hemispheric connectivity.

In summary, astrocytes are indispensable architects of corpus callosum development. State-of-the-art research emphasizes their multifaceted contributions from anatomical remodeling, to guiding axons at the earliest stages and maintaining homeostasis and regulating synaptogenesis in the developing and maturing CC. In disease conditions, astrocytes can also contribute to pathology—reactive astroglia may form scars in the CC after injury or demyelination, and astrocytic dysfunction is implicated in disorders that feature callosal myelination defects (Bonetto et al., 2021; Gzielo and Nikiforuk, 2021; Morcom et al., 2021b). Conversely, enhancing astrocytic support functions is being explored therapeutically (e.g., transplanting astrocyte progenitors to promote regeneration) (Sullivan et al., 2020). Overall, astrocytes in the CC are not mere supporters of neuronal development and functions, but they are active participants, ensuring that callosal axons find their paths, interacting with neurons through metabolic support and signaling molecules.

## Oligodendrocytes in CC development

Oligodendrocytes are the myelin-forming glia of the central nervous system, crucial for the maturation and plasticity of axonal networks and the CC. Oligodendrocyte precursor cells (OPCs) arise from the ventricular and subventricular zones during mid-gestation and migrate later into the developing corpus callosum (Mitew et al., 2014). In rodents, OPCs start colonizing the CC around late embryonic stages; in humans, OPCs are present in the fetal CC and expand postnatally as myelination ramps up. The migration, proliferation, and differentiation of OPCs in the callosal region are tightly regulated by multiple signaling pathways in the developing brain (Galichet et al., 2021; Buchanan et al., 2023; Akinlaja and Nishiyama, 2024). The pathways and molecular actors affecting OPC development are listed in Table 2.

**TABLE 2:** Crucial signaling pathways for CC development in the fetal brain.

Signaling pathway	Function	Pathway disturbance outcome	References
Sonic hedgehoge (Shh)	Expressed in the ventral forebrain, stimulates OPC specification and migration to the CC, promoting early proliferation to ensure a sufficient precursor pool.	Loss of Shh signaling can reduce OPC numbers and delay callosal myelination.	(Nery et al., 2001; Ferent et al., 2013)
Platelet-derived growth factor (PDGF)	In the developing corpus callosum, neurons and astrocytes secrete PDGF-A, which binds PDGFRα on OPCs to promote their survival and proliferation ahead of myelination.	Mice lacking PDGF-A have significantly fewer oligodendrocytes and hypomyelinated callosal.	(Ellison et al., 1996; Fruttiger et al., 1999)
Fibroblast growth factor (FGF-2)	Plays an inhibitory role in the differentiation of oligodendrocyte progenitors into mature oligodendrocytes during remyelination in the adult central nervous system.	Elevated levels of FGF2 promoted progenitor proliferation, while reduced FGF2 activity (using neutralizing antibodies) enhanced progenitor differentiation. Therefore, FGF2 seems to favor the proliferation of oligodendrocyte progenitor cells (OPCs) rather than their differentiation into oligodendrocytes.	(Armstrong et al., 2002)
Wnt/β-Catenin	High Wnt activity in the corpus callosum maintains OPCs in an undifferentiated, proliferative state; its downregulation is required for maturation and myelination.	Wnt/β-catenin signaling regulates myelination timing: overactivation inhibits oligodendrocyte differentiation, while loss leads to premature maturation and reduced OPC numbers.	(Feigenson et al., 2009; Liu et al., 2022; Zhang W. et al., 2023)
Notch1	Notch signaling delays oligodendrocyte maturation to preserve precursors, ensuring sustained production during CC growth.	Disruption of Notch1 signaling impairs the balance between OPC maintenance and differentiation, leading to either premature myelination with precursor depletion or blocked maturation and hypomyelination.	(Zhang et al., 2009)
Bone morphogenetic protein (BMP)	Opposes oligodendrocyte maturation. BMPs push glial progenitors toward an astrocyte fate at the expense of oligodendrocytes.	Elevated BMP impairs myelination, while its inhibition promotes remyelination, marking it as a negative regulator.	(Sabo et al., 2011)
Thyroid hormone (TH)	Thyroid hormones (T3/T4) are essential for timely myelination, promoting OPC differentiation and myelin gene expression; their replacement can rescue deficits in developing animals.	Hypothyroid conditions (e.g., congenital hypothyroidism) often lead to delayed callosal myelination and cognitive deficits.	(Luongo et al., 2021)
Neuregulin-1/ErbB	Neuronal NRG1 signals via ErbB receptors on oligodendrocyte lineage cells to regulate OPC survival, migration, and myelination in the corpus callosum, targeting active axons.	Proper NRG1-ErbB signaling is crucial for callosal myelination; low NRG1 causes hypomyelination, while excessive ErbB2/3 activity leads to abnormal myelin and oligodendrocyte stress.	(Hu et al., 2021)
Extracellular matrix (ECM) and integrins	In the corpus callosum, ECM molecules like laminin and fibronectin guide OPC migration via integrin receptors and help trigger differentiation upon axon contact. Laminin promotes oligodendrocyte progenitor cell differentiation	Abnormal ECM (like in congenital muscular dystrophy with brain involvement) can lead to OPC misplacement or delayed myelination in the CC.	(Relucio et al., 2009)

Once OPCs have migrated and proliferated in the corpus callosum, they differentiate into mature oligodendrocytes. This differentiation is marked by the expression of myelin proteins (e.g., MBP, PLP, MOG) and by the wrapping of oligodendrocyte processes around axons to form myelin internodes. Myelination in the CC starts perinatally in many mammals and accelerates during infancy. In humans, myelination proceeds roughly from posterior to anterior (the splenium myelinates before the genu), continuing into the second and third decades of life (Mount and Monje, 2017; Kuhn et al., 2019; Hughes, 2021).

Oligodendrocytes provide metabolic and trophic support to neurons by delivering lactate to axons and buffering ions, thus helping maintain axonal homeostasis (Kuhn et al., 2019; Kennedy et al., 2025). If oligodendrocyte development is perturbed, axonal caliber and survival can be affected. For example, in mouse models where oligodendrocyte generation is blocked or significantly delayed, callosal axons show stunted growth and some degenerate over time, demonstrating that oligodendrocytes are essential partners in axon integrity (Simons and Nave, 2016; Simons et al., 2024).

Recent research highlights the role of oligodendrocytes in activity-dependent plasticity. Rather than being static, myelination by oligodendrocytes can adapt in response to neuronal activity, a phenomenon termed adaptive myelination (Gibson et al., 2014; Knowles et al., 2022). Studies show that learning new skills or exposing animals to enriched environments induces additional myelin formation in relevant callosal pathways (Tripathi et al., 2017; Nicholson et al., 2022). This suggests that oligodendrocytes contribute to refining neural circuits even beyond early development. Myelin plasticity may help fine-tune neural signal transmission between hemispheres, thereby affecting functions like language, motor coordination, and cognition that rely on the CC (Xin and Chan, 2020). For instance, increased motor learning in mice leads to heightened oligodendrogenesis and myelination in motor callosal fibers (Simons and Nave, 2016; Simons et al., 2024). A recent review noted that myelin changes can strengthen circuit function underlying learning and memory (Bonetto et al., 2021). Mechanistically, neuronal firing can promote the proliferation and differentiation of nearby OPCs (through glutamatergic signaling and activity-induced astrocytic ATP/K^+^ release), leading to new myelin sheaths on active axons. This plastic myelination is thought to support circuit plasticity by optimizing conduction speed, and its dysregulation could contribute to developmental disorders (Chen et al., 2018; Yang et al., 2020).

Proper oligodendrocyte development in the CC is critical for its formation and adequate inter-hemispheric functions. Hypomyelination of the corpus callosum in infancy can manifest as developmental delay, poor motor coordination, or cognitive deficits. For example, preterm infants with periventricular leukomalacia (white matter injury) often show reduced callosal volume and later motor and cognitive impairments; this is due in part to loss or injury of OPCs during a vulnerable perinatal period (Back, 2017). Genetic disorders affecting oligodendrocytes, such as leukodystrophies, typically involve the corpus callosum prominently (diagnosed as a thinned CC or abnormal T2 signal on MRI) and lead to severe neurological symptoms (van der Knaap and Bugiani, 2017). Diffusion tensor imaging studies in schizophrenia and autism have found subtle callosal white matter microstructural abnormalities, suggesting that oligodendrocyte dysregulation may contribute to the connectivity changes in these conditions (Haigh et al., 2019; Faraji et al., 2023). In addition, research in multiple sclerosis has raised interest in enhancing oligodendrocyte regeneration; several experimental therapies that promote OPC differentiation (e.g., the antihistamine clemastine and other remyelinating agents) are being explored and associated with developmental myelination disorders (Yamazaki and Ohno, 2025).

In summary, oligodendrocytes are central to the formation, maturation, and functions of the CC. Through tightly regulated developmental programs, oligodendrocyte lineage cells populate the CC, wrap axons with myelin, and support axonal function. Advances in developmental neuroscience and glial biology continue to shed light on the molecular mechanisms regulating oligodendrogenesis and myelination in the CC, offering insights into how disruptions in these processes lead to neurodevelopmental disorders and how activity or experience can influence callosal connectivity via adaptive myelination.

## Microglia in CC development

Microglia are the resident immune cells of the central nervous system and emerge early during embryonic development. Unlike astrocytes and oligodendrocytes, that originate from the neuroectoderm, the microglia derives from the yolk sac cells progenitors colonizing the embryonic brain before the formation of the blood-brain barrier (Ginhoux et al., 2010). In mice, microglia begin colonizing the brain around embryonic day 9.5, and by mid-gestation, they are widely distributed in developing white matter tracts, including the developing corpus callosum (Alliot et al., 1999; Thion et al., 2018).

Microglia play essential roles in regulating axon guidance, structural organization, and synaptic remodeling. In early stages, microglia help organize callosal axons into compact bundles by promoting fasciculation (Fujita and Yamashita, 2021). During early brain development, microglia facilitate axon fasciculation through a limited set of well-defined molecular pathways. The adaptor protein DAP12 (TYROBP) mediates microglial signaling required for callosal axon bundling, as its loss causes marked defasciculation (Pont-Lezica et al., 2014). In parallel, neuronal Netrin-G1 and microglial NGL1 (LRRC4C) interactions recruit microglia to developing axon tracts, while microglia-derived IGF-1 provides trophic support that stabilizes and promotes axonal growth (Fujita et al., 2020). Additionally, microglia secrete thrombospondins and remodel the extracellular matrix via MMP-2/9, creating a permissive environment for axon bundling. Together, these pathways establish the main molecular framework through which microglia orchestrate axon fasciculation during central nervous system (CNS) development (Squarzoni et al., 2014; Fujita et al., 2020). Experiments using microglia-deficient models, such as Pu.1 knockout mice, have shown that the absence of microglia leads to defasciculated and misrouted callosal axons (Pont-Lezica et al., 2014). This suggests that microglia not only interact physically with growing axons but also secrete trophic factors that help maintain axonal alignment, such as IGF-1 and BDNF to promote axon growth, and adhesion molecules like ICAM-1, VCAM-1, and integrins to stabilize and fasciculate developing axon bundles (Carlstrom et al., 2011; Guo et al., 2018; Constantin et al., 1999).

Microglia play a critical role in refining neural circuits through synaptic remodeling, selectively eliminating weak or inactive synapses to optimize connectivity. This process involves molecular mechanisms such as the complement cascade (e.g., C1q, C3) and receptors including CR3 and TREM2, which help microglia identify and engulf unnecessary synapses (Neniskyte and Gross, 2017; Ball et al., 2022; Guedes et al., 2022). In the context of corpus callosum development, synaptic pruning by microglia ensures that functionally relevant interhemispheric connections are maintained, contributing to efficient neural communication between hemispheres. Disruptions in microglial synaptic remodeling can lead to abnormal callosal connectivity, such as overconnectivity or hypoconnectivity, which have been linked to neurodevelopmental disorders including autism spectrum disorder and schizophrenia (Luo and Wang, 2024). In addition, microglia have been implicated in axonal pruning in other brain regions or developmental stages, evidence for direct microglial removal of callosal axons during corpus callosum formation is limited and remains an area of ongoing investigation.

In the postnatal period, microglia refine callosal circuitry through activity-dependent synaptic remodeling. Astrocyte-derived molecules such as IL-33 and TGF-β modulate microglial behavior by enhancing their capacity to prune inactive synapses, thus supporting efficient neural signaling between hemispheres (Vainchtein et al., 2018; Zhang et al., 2025).

Microglia also influence oligodendrocyte progenitor development. In early postnatal life, they organize surplus OPCs, which help regulate the density and spacing of myelinating cells; consistent with this, Nemes-Baran et al. (2020) demonstrated that fractalkine receptor-deficient mice exhibit reduced microglial engulfment leading to an increased number of oligodendrocytes but paradoxically reduced myelin thickness, suggesting that microglial regulation is critical not just for OPC number but also for their maturation and myelination efficiency (Nemes-Baran et al., 2020).

Microglia also release factors such as IGF-1 that promote oligodendrocyte maturation and myelin production. Under pathological conditions, however, activated microglia can secrete inflammatory cytokines that impair OPC differentiation and reduce myelination, contributing to white matter injury (Smith et al., 2012; Rusin et al., 2024).

Microglia surveillance throughout the CNS, by clearing apoptotic cells, responding to environmental insults, and maintaining tissue homeostasis, play a major role in CC development and maturation. In the corpus callosum, microglia contribute specifically by shaping axonal architecture, refining synapses, and regulating myelination. Disruption of microglial function, whether through genetic or environmental factors, impaired callosal developmental processes and lead to callosal malformations or other connectivity defects (Santos and Fields, 2021; McNamara et al., 2023).

## Neuron-glia crosstalk in corpus callosum development

Astrocytes, oligodendrocytes, and microglia collaborate to orchestrate corpus callosum formation through tightly regulated molecular and cellular mechanisms. Midline astrocytes act as guideposts, forming the glial wedge and zipper glial structures that secrete guidance cues such as Slit, Netrin-1, and growth factors (FGF8, IGF-1) to direct pioneer callosal axons, while providing scaffolding and metabolic support via GLUT1/MCT1-mediated lactate shuttling and EAAT/Kir4.1-mediated ionic homeostasis. Oligodendrocyte precursor cells (OPCs) migrate into the developing CC under the influence of growth factors, chemokines, and neuronal activity, differentiating into myelinating oligodendrocytes that express MBP, PLP, and MOG; these cells not only enable efficient action potential conduction but also support axonal metabolism and activity-dependent plasticity through adaptive myelination. Microglia further refine CC circuitry by promoting axon fasciculation via DAP12, NGL1/Netrin-G1, and IGF-1, pruning excess axons and synapses through complement signaling (C1q/C3–CR3/TREM2) and modulating oligodendrocyte maturation, with astrocyte-derived IL-33 and TGF-β shaping microglial activity. Together, these glial populations integrate structural, metabolic, and signaling functions to ensure precise axon guidance, myelination, and synaptic connectivity, and disruptions in any of these pathways can lead to callosal malformations or connectivity deficits implicated in neurodevelopmental disorders (Gobius et al., 2016; Fujita et al., 2020; Pont-Lezica et al., 2014).

As discussed above, the development of the CC relies on precisely coordinated communication between neurons and glial cells, which together regulate axon guidance, myelination, and synaptic remodeling. This intercellular dialogue occurs through the dynamic exchange of molecular signals (ligands, receptors, and intercellular junctions) that direct the spatial and temporal patterning of CC development (Table 3). Understanding the functional consequences of these pathways requires examining not only the molecules involved but also their sources, targets, and downstream effects.

**TABLE 3:** Key integrated pathways.

Key integrated pathways	Function	Effect of the disturbance	References
Netrin-1/DCC (neuron–glia signaling)	Netrin-1 from midline glia guides callosal axons via DCC receptors and regulates astroglial arrangement, coordinating neural and glial development.	In Netrin-1 or DCC knockout mice, callosal axons fail to reach the midline, leading to misrouting or incorrect commissure formation.	(Morcom et al., 2021b; Fothergill et al., 2014)
Slit/Robo (astrocyte–neuron signaling)	Midline astroglia secrete Slit to repel callosal axons via Robo receptors after crossing, creating a one-way gate with Netrin attracting axons initially; Robo1/2 also modulates this switch for precise axon guidance.	Without Slit-Robo signaling, axons stall or misroute at the midline; Slit2 or Robo1/2-deficient mice show axon clumping and septal misrouting.	(Unni et al., 2012)
Neuregulin-1/ErbB (neuron–oligodendrocyte signaling)	NRG1 on callosal axons binds ErbB2/3 on OPCs to regulate their migration, survival, and myelination; larger axons express more NRG1, promoting differentiation. Astrocytes modulate this process, while timing of ErbB signaling is critical, as overactivation causes premature differentiation or stress.	Elevated NRG1 enhances myelination, while its loss causes thinner or delayed myelin; excessive ErbB signaling leads to dysmyelination, underscoring the need for balanced NRG1-ErbB signaling.	(Taveggia et al., 2008)
CX3CL1/CX3CR1 (neuron–microglia signaling)	Neuronal CX3CL1 binds microglial CX3CR1 to regulate microglial activity, suppress inflammation, and guide synapse pruning; in the corpus callosum, it controls microglial numbers and phagocytosis for proper development.	CX3CR1-deficient mice show impaired microglial pruning and cortical overconnectivity. Neuronal fractalkine attracts microglia for synapse remodeling and injury response, but disrupted signaling can cause microglial dysfunction, affecting corpus callosum development.	(Irfan et al., 2022; Luo et al., 2019)
Astrocyte–microglia and astrocyte–neuron signaling	Astrocytes and microglia likely interact in a trophic and immune-modulatory manner during development. Astrocytes release cytokines (such as TGF-β and IL-33) that regulate microglial activation. Astrocytes are crucial for synaptic transmission, ion balance, and neurotransmitter regulation, all of which are necessary for proper neuronal function.	Defasciculation (loss of organized bundle formation) of dorsal callosal axons.	(Pont-Lezica et al., 2014; Vainchtein et al., 2018)

A well-characterized pathway is the Slit-Robo signaling axis, essential for midline navigation. Midline astrocytes in the glial wedge and indusium griseum glia secrete Slit2, which binds to Robo1/2 receptors expressed on cortical callosal axons. This interaction exerts a driving force that channels axons toward and across the interhemispheric fissure, preventing them from straying into inappropriate regions (Unni et al., 2012; Gonda et al., 2020). In parallel, radial glia and ventral midline cells produce Netrin-1, which binds to DCC receptors on the same neurons, acting as an attractive signal (Unni et al., 2012; Fothergill et al., 2014). Together, these cues form a molecular corridor guiding axons to and through the midline. Robo expression increases after crossing, sensitizing axons to Slit-mediated repulsion and preventing re-crossing (Fothergill et al., 2014).

Additional astrocyte-secreted factors, including fibroblast growth factor 8 (FGF8) and insulin-like growth factor 1 (IGF-1), further contribute to axonal extension and navigation by modulating the extracellular matrix and enhancing axonal responsiveness to guidance cues (Dyer et al., 2016; Zhang W. et al., 2023). Neuron-to-glia signaling also plays a crucial role in the development of oligodendrocytes and myelination. Neurons express membrane-bound Neuregulin-1 (NRG1), which interacts with ErbB2/3 receptors on OPCs. This interaction promotes OPC survival, migration, and terminal differentiation (Kim et al., 2003; Hu et al., 2024). The magnitude of NRG1 signaling is proportional to neuronal activity, allowing active axons to be preferentially myelinated. Astrocytes influence this pathway indirectly by releasing extracellular modulators that affect ErbB receptor expression and NRG1 availability.

Microglia support corpus callosum formation by interacting with growing axons, shaping axonal architecture, and maintaining a permissive environment for midline crossing during fetal development. After birth, microglia contribute to the refinement of callosal projections by participating in synaptic remodeling and final inter-hemispheric connectivity. Neurons release CX3CL1 (fractalkine), which binds CX3CR1 receptors on microglia, regulating their surveillance and phagocytic activity, including synaptic pruning (Paolicelli et al., 2014; Pawelec et al., 2020; Wang et al., 2021). Astrocytes also signal via interleukin-33 (IL-33), promoting microglial phagocytosis of underactive synapses in an activity-dependent manner, thus reinforcing active synapses and eliminating superfluous ones (Vainchtein et al., 2018; Han R. T. et al., 2023).

Crosstalk involving activity-dependent feedback loops further regulates glial function. Neuronal firing elevates extracellular ATP and potassium, which are detected by astrocytes and OPCs. These glial cells respond by releasing lactate, growth factors, or pro-differentiation signals that support axon migration and promote myelination (Chen et al., 2024).

Recently, DRAXIN, a secreted chemorepulsive guidance protein, has been shown to play a critical role in CC formation by regulating the astroglial-dependent remodeling of the interhemispheric fissure that precedes axon crossing (Morcom et al., 2021a). In mice, DRAXIN is expressed by dorsal radial glia and emerging midline zipper glia (MZG progenitors) and interacts genetically and biochemically with DCC to modulate callosal axon trajectories (Islam et al., 2009). Loss of Draxin disrupts early steps of IHF remodeling, namely MZG specification, radial glial process extension, and somal translocation, which result in an enlarged, leptomeningeal-filled fissure and failure of pioneer axons to cross (Morcom et al., 2021a). Thus, DRAXIN functions upstream of Slit–Robo and Netrin-DCC signals by establishing the astrocytic scaffold necessary for these cues to guide axons across the midline.

Bidirectional signaling among neurons, astrocytes, oligodendrocytes, and microglia thus forms a coordinated network guiding the successive ontogenesis of the CC, from initial pathfinding and axonal elongation to glial anatomical remodeling, myelin wrapping, and synaptic culling. Disruptions in these communication pathways have been implicated in multiple neurodevelopmental disorders, including autism spectrum disorder, schizophrenia, and congenital callosal agenesis (Szepesi et al., 2018). A mechanistic understanding of these interactions not only clarifies the cellular basis of CC development but also points to potential therapeutic targets for correcting or preventing interhemispheric connectivity deficits.

## Callosal developmental malformations in human disorders and animal models

Experimental models, particularly genetically modified mice, have been pivotal in elucidating the molecular and cellular mechanisms underlying CC development. By targeting specific genes involved in axon guidance, midline patterning, glial development, and synaptic remodeling, these models replicate key features of human callosal malformations and provide insights into conserved developmental pathways. Many gene deletions in mice result in partial or complete callosal agenesis, and corresponding human mutations frequently lead to similar neurodevelopmental anomalies (Gavrish et al., 2024).

One of the best-characterized models involves the deletion of *Dcc* (Deleted in Colorectal Carcinoma), a receptor for the guidance molecule Netrin-1. Mice lacking Dcc exhibit complete agenesis of the CC, with axons failing to cross the midline and instead forming Probst bundles (Fothergill et al., 2014; Morcom et al., 2021b). In humans, heterozygous mutations in *DCC* cause congenital syndrome and partial callosal agenesis, emphasizing the translational value of knock-out mouse models (Marsh et al., 2017, 2018).

**FIGURE 2:**
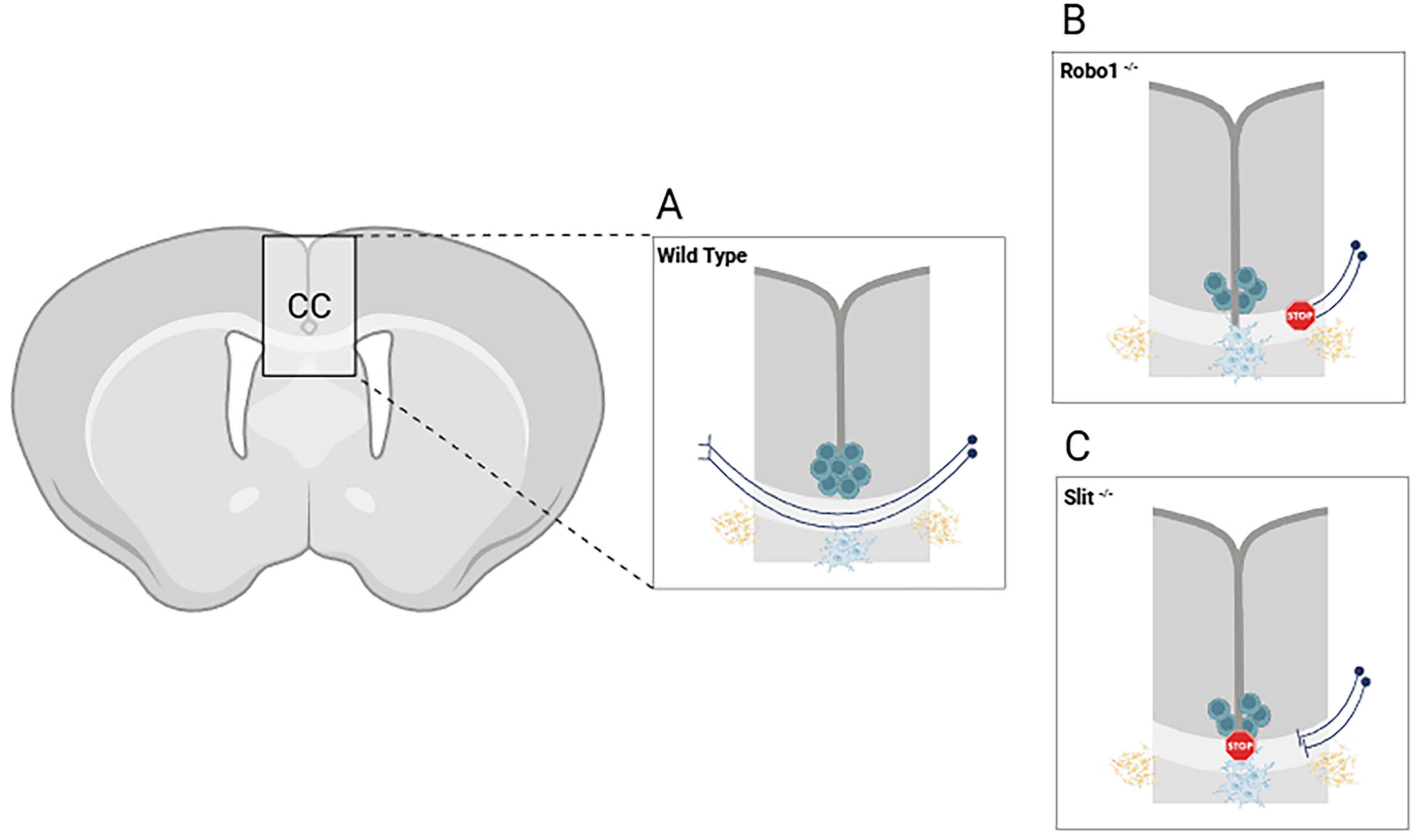
Schematic representations of CC developmental malformations in two genetic mouse models. **(A)** The successful development of the CC in wild type brain is mediated by signaling interactions between glial structures and neurons (i.e., Slit1-Robo1 pathway). **(B)** Knock-out mice carrying mutations in the Robo1 receptor gene display callosal malformation due to impaired neuronal responsivity to the Slit1 guidance factor. **(C)** Knock-out mice lacking the glia-derived Slit1 guidance factor exhibit similar callosal malformations as in the Robo1 mutant, although resulting from disrupted ligand secretion by the glial structures. Created in BioRender (https://app.biorender.com/citation/6904bbd3cf3d6e0fe32c2298).

Similarly, knockout of Ntn1, encoding Netrin-1 protein, also leads to severe callosal pathfinding defects in mice, reinforcing the essential nature of the Netrin-DCC axis in midline crossing (Sagi-Dain et al., 2020). Double knockout of Robo1/2, the receptors for Slit proteins, results in aberrant axonal trajectories and misrouting of callosal fibers into subcortical regions (Andrews et al., 2006; Fouquet et al., 2007; Tamada et al., 2008). Mice with disrupted Slit2 also show axons accumulating near the midline without successful crossing, recapitulating features observed in humans with agenesis due to midline glial dysfunction and similar mutations (Long et al., 2004; Figure 2).

The X-linked L1CAM gene, encoding an axonal cell adhesion molecule, is another major player in callosal development. L1cam-deficient mice show the absence of the CC and abnormal axon fasciculation. In humans, mutations in L1CAM cause CRASH syndrome (Corpus callosum agenesis, Retardation, Adducted thumbs, Spastic paraplegia, and Hydrocephalus), a well-documented X-linked disorder linked to glial tissue and its surrounding extracellular environment (Fransen et al., 1995; Yamasaki et al., 1997).

Knockouts affecting midline glia-specific morphogens also result in callosal abnormalities. For example, mice lacking Fgfr1 or Gli3, involved in the glial wedge formation and dorsal telencephalon patterning, develop agenesis or hypoplasia of the CC (Magnani et al., 2014; Gobius et al., 2016). These models underscore the importance of astrocyte-mediated midline remodeling in creating a permissive path for axons.

Mutations in genes affecting oligodendrocyte function and myelination also lead to structural anomalies in the CC. Mice deficient in Plp1 or Mbp show delayed or incomplete callosal myelination (Arinrad et al., 2023). In humans, leukodystrophies such as Pelizaeus-Merzbacher disease (linked to PLP1 mutations) and metachromatic leukodystrophy (linked to ARSA) feature callosal thinning and signal changes visible on MRI (Groeschel et al., 2011; Laukka et al., 2014). Hypomyelination and disrupted oligodendrocyte development during key developmental windows can thus significantly affect callosal integrity (Anderson et al., 2018).

Disruptions in microglial signaling also result in callosal defects. Cx3cr1-knockout mice exhibit impaired synaptic pruning and excess dendritic spines, alongside transient delays in callosal refinement (Paolicelli et al., 2011). Mice with maternal immune activation (MIA) or deletions in complement components (e.g., C1q, C3, Trem2) show altered synaptic density and impaired microglial pruning, leading to aberrant interhemispheric connectivity (Filipello et al., 2018; Yan et al., 2024). These immune-related findings have been linked to risk factors for neurodevelopmental disorders such as autism and schizophrenia.

Furthermore, knockout or knockdown of IL33, an astrocyte-derived cytokine essential for microglia-mediated synapse elimination, impairs developmental pruning and results in circuit hyperconnectivity. These phenotypes parallel features observed in human ASD brains, which often show increased spine density and abnormal CC morphology (Vainchtein et al., 2018).

In addition to targeted knockouts, naturally occurring inbred strains have demonstrated genetic backgrounds predisposing to callosal agenesis. For example, the BTBR T^+^ Itpr3^–^/^–^ mouse strain exhibits a complete absence of the CC, and attendant forebrain connectivity deficits, making it a widely used model for studying idiopathic agenesis and its behavioral and metabolic correlates (Vega-Pons et al., 2017; Martin et al., 2021; Morcom et al., 2021a; Viscomi et al., 2025). Similarly, BALB/c mice display variable thinning or partial absence of the rostral CC, correlating with altered interhemispheric connectivity although less penetrant (Wahlsten, 1989; Wahlsten and Schalomon, 1994; Bohlen et al., 2012). These inbred models underscore that polygenic and strain-specific factors, beyond single-gene disruptions, can profoundly impact midline glial patterning and axon guidance in the developing CC.

Collectively, mouse models have provided crucial evidence that both neuronal and non-neuronal genes regulate CC development. The parallel occurrence of similar phenotypes in humans with orthologous gene mutations (e.g., DCC, L1CAM, PLP1) validates these models, thus supporting their use in dissecting the cell-type-specific roles of glia and neurons. Ongoing refinement of conditional and cell-type-specific knockout strategies will further delineate the contribution of each glial population to specific stages of CC ontogenesis.

## Emerging tools and translational perspectives

A comprehensive understanding of CC development increasingly relies on tools capable of dissecting the specific roles of glial and neuronal cells. While classical studies often focused on axonal pathfinding, newer experimental approaches have shifted attention toward how astrocytes, oligodendrocytes, and microglia orchestrate axon guidance, myelination, and synaptic remodeling.

Conditional knockout mouse models have become indispensable for clarifying cell-type-specific functions. Cre-loxP systems using promoters such as *Aldh1l1-Cre* for astrocytes, *Olig2-Cre* for oligodendrocyte lineage cells, and *Cx3cr1-Cre* for microglia allow for targeted gene manipulation (Livet et al., 2007; Hu et al., 2020). For example, astrocyte-specific deletion of *Fgfr1* or *Gli3* disrupts glial wedge formation and leads to callosal agenesis (Gobius et al., 2016; Morcom et al., 2021b). Likewise, *Cx3cr1-Cre* mice have been used to show that altered microglial activity during prenatal and early postnatal periods results in callosal miswiring and overconnectivity (Hoshiko et al., 2012; Sahasrabuddhe and Ghosh, 2022).

*In vivo* imaging techniques such as time-lapse two-photon microscopy and magnetic resonance imaging (MRI) are being adapted to monitor glial behavior and CC integrity in real-time. Labeling of glial subpopulations with fluorescent reporters (e.g., *Sox10-GFP* for oligodendrocytes, *Cx3cr1-GFP* for microglia) enables the visualization of cell migration, axon interaction, and morphological remodeling in developing brains. Diffusion tensor imaging (DTI) also allows the correlation of microstructural white matter changes with genetic or environmental alterations affecting glial cell populations (Zhang B. et al., 2014).

Single-cell and spatial transcriptomics provide further resolution into glial diversity and function within the developing CC. These techniques allow the identification of transcriptional states linked to distinct roles in axon guidance, myelination, or pruning. For instance, scRNA-seq datasets from developing mouse brains have revealed temporally shifting microglial gene signatures associated with phagocytosis, complement activation, and cytokine signaling. Astrocyte and OPC populations likewise exhibit unique gene expression profiles correlated with midline signaling, extracellular matrix remodeling, and metabolic support to the neuronal pathfinding and functions (Zhang B. et al., 2014; Li et al., 2019; Allen et al., 2023; Han Z. et al., 2023).

*In vitro* models also offer controlled environments to dissect molecular pathways. Co-culture systems of neurons with glia, especially astrocytes or OPCs, permit functional assays of axon outgrowth, myelination, and synaptogenesis (Shimizu et al., 2011; Roqué and Costa, 2017; Goshi et al., 2020). Application of recombinant proteins (e.g., Slit2, Netrin-1, IL-33, TGF-β) or pharmacological inhibitors (e.g., PDGFR blockers, Notch antagonists) further refines these models. Additionally, patient-derived iPSC lines can be differentiated into glial subtypes to evaluate the functional impact of disease-associated variants on CC-relevant processes (Frati et al., 2018).

Recent advances in brain organoids and assembloids technologies allow partial modeling of interhemispheric connections and glial development in a human cellular context (Wu and Nowakowski, 2025). Although callosum-like axonal tracts remain incomplete in these systems, improvements in regional patterning and glial differentiation protocols are enhancing their translational value. Transplantation of oligodendrocyte progenitors or astrocyte precursors into mouse brains or cerebral organoids is also being explored to test the capacity of glial cells to support or restore callosal function (Luciani et al., 2024).

Overall, the integration of conditional genetics, live imaging, transcriptomics, and iPSC-based modeling is evolving the study of glial cells into cell-specific functions and signaling affecting CC development. These approaches not only deepen our understanding of cellular interactions in the developing forebrain but also help establish mechanistic links to human disorders marked by callosal dysgenesis. Moving forward, these models and tools are essential for uncovering cell-type-specific vulnerabilities and potential therapeutic strategies targeting glial dysfunction.

## Concluding remarks and future directions

**FIGURE 3:**
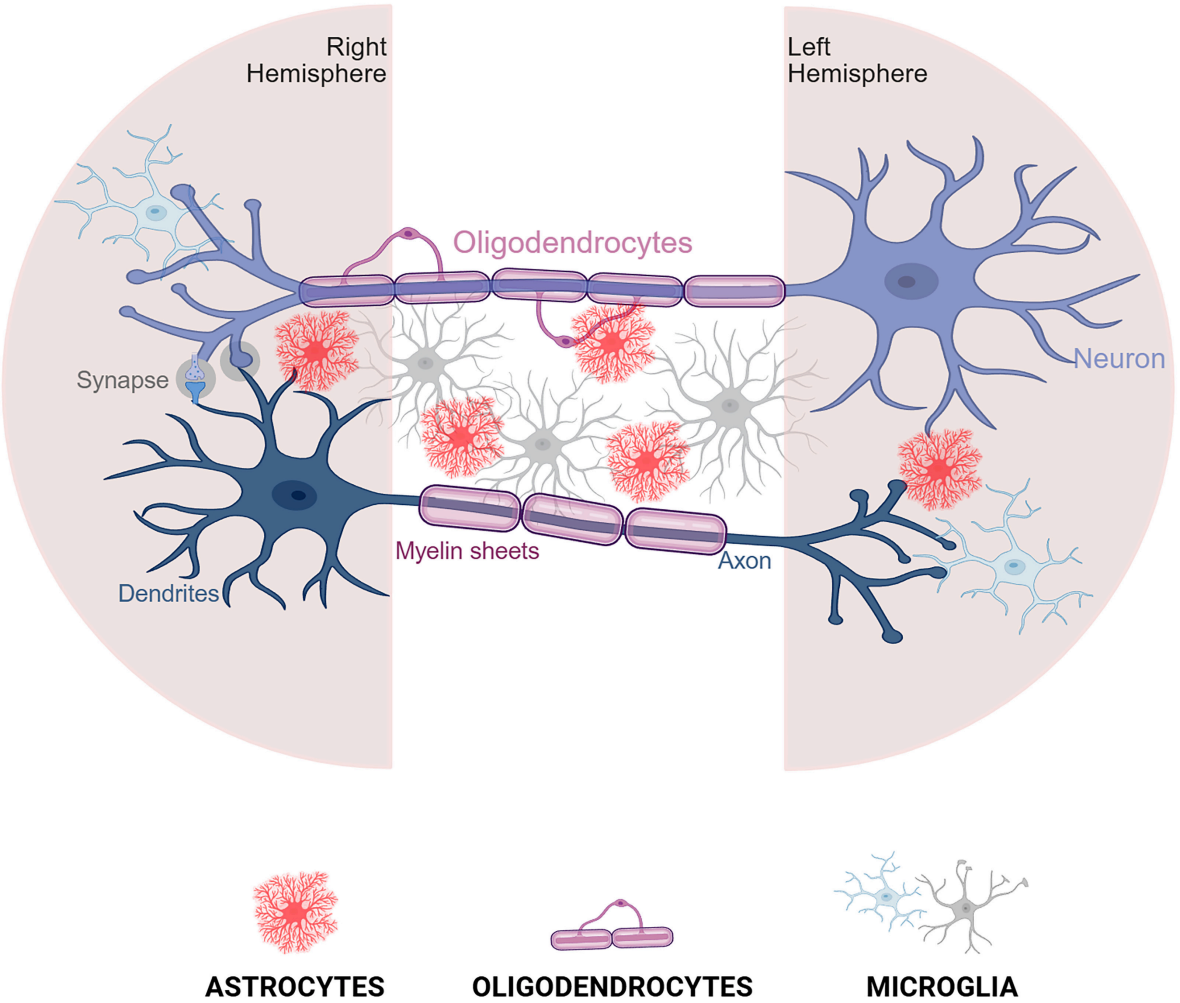
Cooperation between microglia, astrocytes, and oligodendrocytes in the postnatal brain. This schematic illustrates the dynamic interactions where microglia mediate synaptic pruning and immune surveillance, astrocytes support neuronal health and modulate synapse maturation, and oligodendrocytes facilitate myelination. Together, these glial cells coordinate to maintain neural circuit integrity and promote proper development and function of the corpus callosum. Created in BioRender (https://app.biorender.com/citation/6904bbd3cf3d6e0fe32c2298).

The development of the corpus callosum involves a complex orchestration of cellular interactions, where glial cells function not merely as passive support elements but as active, essential participants in every phase of interhemispheric connectivity, contributing to the growth and survival of callosal neuronal projections (Figure 3). Overall, the evidence presented throughout this review affirms that callosal development is not a neuron-centric process but rather a cooperative venture involving diverse glial populations.

Comparative studies in rodents and humans reveal both conserved pathways and species-specific adaptations in CC development. Importantly, environmental factors such as inflammation, hypoxia, or endocrine disruption interact with glial programs to influence outcomes, underlining the plasticity and vulnerability of this system.

The convergence of animal models and human data strongly supports the hypothesis that defects in glial function contribute to various callosal pathologies, from congenital agenesis to white matter disorders associated with neurodevelopmental syndromes. These pathologies often involve disruptions in fetal brain remodeling processes, and/or in neuron-glia signaling pathways such as Netrin-DCC, Slit-Robo, Neuregulin-ErbB, and CX3CL1-CX3CR1, suggesting a central role for glia in modulating axonal guidance, synaptic refinement, and structural maturation.

Despite significant advances, critical questions remain unanswered. The specific molecular programs that define regional glial heterogeneity within the CC, the temporal coordination of neuron-glia communication across developmental windows, and the degree to which these mechanisms can be targeted for therapeutic intervention remain active areas of research. The functional consequences of glial dysfunction in neuropsychiatric disorders also warrant deeper investigation. Future directions include the integration of multimodal single-cell datasets (transcriptomic, proteomic, etc.) with cell-type-specific gene editing tools to map causal mechanisms. Longitudinal studies in human cohorts, supported by iPSC and organoid/assembloid models, are expected to refine translational applications.
